# Recent Advances on Innovative Materials from Biowaste Recycling for the Removal of Environmental Estrogens from Water and Soil

**DOI:** 10.3390/ma15051894

**Published:** 2022-03-03

**Authors:** Elisabetta Loffredo

**Affiliations:** Dipartimento di Scienze del Suolo, della Pianta e degli Alimenti, Università degli Studi di Bari Aldo Moro, 70126 Bari, Italy; elisabetta.loffredo@uniba.it; Tel.: +39-080-5442282

**Keywords:** biochar, hydrochar, digestate, endocrine disruptor, estrogen, biosorbent, adsorption, water decontamination, soil remediation

## Abstract

New technologies have been developed around the world to tackle current emergencies such as biowaste recycling, renewable energy production and reduction of environmental pollution. The thermochemical and biological conversions of waste biomass for bioenergy production release solid coproducts and byproducts, namely biochar (BC), hydrochar (HC) and digestate (DG), which can have important environmental and agricultural applications. Due to their physicochemical properties, these carbon-rich materials can behave as biosorbents of contaminants and be used for both wastewater treatment and soil remediation, representing a valid alternative to more expensive products and sophisticated strategies. The alkylphenols bisphenol A, octylphenol and nonylphenol possess estrogenic activity comparable to that of the human steroid hormones estrone, 17β-estradiol (and synthetic analog 17α-ethinyl estradiol) and estriol. Their ubiquitous presence in ecosystems poses a serious threat to wildlife and humans. Conventional wastewater treatment plants often fail to remove environmental estrogens (EEs). This review aims to focus attention on the urgent need to limit the presence of EEs in the environment through a modern and sustainable approach based on the use of recycled biowaste. Materials such as BC, HC and DG, the last being examined here for the first time as a biosorbent, appear appropriate for the removal of EEs both for their negligible cost and continuously improving performance and because their production contributes to solving other emergencies, such as virtuous management of organic waste, carbon sequestration, bioenergy production and implementation of the circular economy. Characterization of biosorbents, qualitative and quantitative aspects of the adsorption/desorption process and data modeling are examined.

## 1. Introduction

One of the most important paradigms of the current period is the preservation of the environment. In recent years, in response to the growing global demand for energy and, at the same time, as a solution to the problem of the enormous mass of organic waste produced annually by agricultural, industrial and municipal activities, various innovative technologies have been implemented to convert biowaste in bioenergy. In addition to gaseous and liquid fuels, these processes generate large quantities of solid carbon-rich coproducts and byproducts, such as biochar (BC), hydrochar (HC) and digestate (DG), which are suitable for various agricultural and environmental applications. In agriculture, these materials are primarily used as multifunctional amendments to restore the organic fertility of soil which is increasingly compromised by intensive and superintensive agriculture [1,2,3]. In soil, these amendments enhance the retention of water and nutrients that are vital for plant growth and production [4]. Another important function of these materials is the immobilization of soil pollutants by adsorption, thus preventing their leaching into groundwater and/or their transport into surface waters. Furthermore, given the recalcitrance of these materials, especially BC and HC, their incorporation into soil allows carbon sequestration for a very long period with a significant reduction in greenhouse gas emissions into the atmosphere [1].

The environmental applications of these materials are based on their excellent ability to behave as adsorbents of a wide variety of both inorganic and organic pollutants [5,6]. This ability is related to their physicochemical properties, such as composition, micromorphology, porosity, functional groups content and degree of aromatization which, in turn, depend on the parameters of the production process, i.e., feedstock, temperature, retention time, pressure and so on. In particular, temperature is a very important parameter of the thermochemical conversion of biomass affecting both the yield of the solid product and its properties and best use. Low temperatures generally prevent a high degree of carbonization and promote the formation of reactive O-containing functional groups that allow the interaction of the material with a wide range of solutes and a consequent more suitable use in agriculture [7]. On the contrary, high temperatures favor a high degree of aromaticity, large specific surface area, low H/C ratio, high C/N ratio and high number of adsorption sites. All these characteristics are indicative of an intense thermal alteration of the raw biomass and suggest a better use of the material for environmental purposes [7]. In any case, in order to select and optimize the applications of these materials, all these properties must be carefully determined [8,9,10]. Characterization data are also essential to evaluate the chemical structure of the material and the possible mechanisms of interaction with pollutants.

Some aspects of individual BC and HC have been recently reviewed [10,11]. In general, these studies considered a broad context of pollutants, including dyes, pharmaceuticals, polycyclic aromatic hydrocarbons PAHs, heavy metals, antibiotics and single EDCs. Furthermore, DG has been studied so far almost exclusively for agricultural use, although recently it has shown an appreciable ability to retain hydrophobic compounds such as the EDCs bisphenol A (BPA) and 4-tert-octylphenol (OP) [12]. It is reasonable to believe that also DG could be used as a biosorbent, possibly after further studies and improvements. Hence, all three materials examined in this review can be considered as innovative tools for wastewater treatment and soil remediation [13,14]. Furthermore, since these biosorbents are essentially a waste of bioenergy technologies, and consequently have an almost zero cost, their exploitation for sustainable environmental depollution seems very interesting and appropriate. In the last decades, world population growth and industrial development have caused a significant increase in the number and quantity of anthropogenic pollutants released into the environment. Among these compounds, a major attack on wildlife and human health is due to a class of contaminants known as endocrine-disrupting chemicals (EDCs) for their proven ability to alter the normal hormonal functions of animals, especially aquatic animals [15]. Both ascertained and suspected EDCs have been detected in human urine, blood and breast milk [16,17,18]. Currently, these compounds are ubiquitous in natural waters, wastewaters, soil, sediments, food and consumer products. EDCs have been widely reported in ecosystems surrounding the most urbanized and industrialized areas [19,20] but have also been detected in remote areas of the globe [21]. A long-term monitoring study based on thousands of samples revealed the presence of BPA, which is one of the most representative EDCs, in freshwater, seawater, freshwater sediments and marine sediments at average levels of 29 ng L^−^^1^, 7 ng L^−1^, 7 ng g^−1^ and <0.03 ng g^−1^, respectively, in Europe, and 5 ng L^−1^, 1 ng L^−1^, 0.7 ng g^−1^ and 1 ng g^−1^, respectively, in North America [22]. In a seasonal monitoring of 14 EDCs, including BPA, in a lagoon located in a coastal area of Southern Italy, single EDCs were detected at concentrations ranging from 132 to 28,000 ng L^−1^ in water and from 0.7 to 155 ng g^−1^ in sediments [23]. In effluents from wastewater treatment plants (WWTPs), BPA has been detected at concentrations up to 370,000 ng L^−1^ [21]. In soil, BPA concentration ranged between <0.01 and 1000 ng g^−1^ in dependence on the amount and type of effluent or waste received [21]. Due to their dangerous effects, EDCs have increasingly attracted the attention of scientists, international organizations, decision-makers and the community [15].

The class of EDCs includes various xenoestrogens, i.e., biologically active synthetic compounds that mimic the activity of the steroid hormone 17β-estradiol. Due to their wide diffusion in the environment, they are also known as environmental estrogens (EEs). Based on their chemical structure, EEs can be grouped as phenolic environmental estrogens (PhEEs) and steroidal environmental estrogens (StEEs). Both groups are often detected in aquatic and terrestrial ecosystems such as rivers, lakes, sea, soil and sediments where they enter through the application, discharge and disposal of urban and industrial effluents, sewage sludge, agro-zootechnical wastes and so on. Wastewater is the main source of EE contamination in the aquatic environment, as the efficiency of EE removal by full-scale WWTPs is quite low [24]. Even in trace amounts, EEs represent a serious risk for aquatic ecosystems and in particular for fish, in which they cause several physiological and reproductive disorders [21].

Contamination of aquatic systems by multiple EEs is frequently detected [19,20], and it has been demonstrated that in such conditions the reproductive disturbance of aquatic fauna can occur even at individual ineffective concentrations [25]. The environmental risks posed by these compounds are also due to their chemical recalcitrance that leads to a long time of persistence in aquatic and terrestrial ecosystems [26]. In the light of all this, it is clear that the environmental consequences of indiscriminate disposal of untreated or not sufficiently decontaminated wastewater and solid waste are of great concern and often go beyond all reasonable expectations.

Various sophisticated and expensive methods are available to remove organic pollutants from aqueous media. Current technologies are mainly based on chemical and physicochemical processes such as flocculation, precipitation–filtration, adsorption on activated carbon, reverse osmosis, advanced chemical and electrochemical oxidation and photocatalytic degradation [27,28], while biological treatments are essentially based on the use of activated sludge. In most cases, these methods fail to completely remove toxic compounds [27,29]. The sorption process plays an important role in wastewater treatment, and well-designed sorption protocols can release high-quality effluent after treatment. The possibility of using coproducts and byproducts of bioenergy technologies to decontaminate wastewater and even soil can certainly be a valid alternative to other complex and less sustainable methods.

For these reasons, the aim of this manuscript is to focus attention on the potential of materials from biowaste recycling to act as low-cost biosorbents in environmental applications according to a modern and sustainable approach that can represent an added value to environmental benefits already achieved with the production of these materials, such as the virtuous recycling of waste in compliance with the principles of the circular economy, the sequestration of carbon, the reduction of climate-altering gas emissions and a significant supply of renewable energy. Recent literature has been included in this review and the majority of references reported are from the last few years.

## 2. Biosorbents from Biowaste Recycling

### 2.1. Biochar

Among the technologies used for bioenergy production there are the thermo-chemical processes of pyrolysis, gasification, flash carbonization, combustion and others [30] (Table 1). Fast pyrolysis, slow pyrolysis and gasification, besides producing gaseous (syngas) and liquid fuels, generate a solid byproduct known as ‘biochar’ or ‘pyrochar’ when obtained by pyrolysis [31]. The common operating conditions used to produce BC are low-moisture-containing feedstock, temperatures ranging from 300 to 800 °C, very limited oxygen atmosphere and retention time usually greater than 0.5 h [31]. The raw biomass used to feed the process usually originates from forestry, agriculture, food processing and the organic fraction of municipal solid waste (OFMSW). In Mediterranean countries, the substrate for BC production consists mainly of vineyard residues, olive tree pruning, orchard cuttings, wood chips, OFMSW and sewage sludges [32]. Other biowastes used to produce BC are sawdust, corn cobs, rice husks, coconut shells, residues from coffee preparation and so on [32].

BC is a stable carbonaceous material with a very high carbon content (>60%), and therefore its production significantly contributes to carbon sequestration and, consequently, to climate change mitigation [34]. Due to BC recalcitrance, its residence time in soil can be several centuries [35]. The composition and properties of BC strongly depend on the type of feedstock and on the pyrolysis parameters, primarily temperature [7].

When incorporated into soil, BC adds an important carbon pool that improves the physical, chemical and biological properties of soil, especially in the cases of degraded soil, sandy soils and soils having a very low content of water and nutrients. In addition to improving soil fertility, BC can prevent the movement and leaching of contaminants in soil due to its remarkable retention capacity [10]. Other interesting and innovative applications of BC concern the remediation of soil and water contaminated by inorganic pollutants such as heavy metals and organic pollutants such as polyaromatic hydrocarbons, agrochemicals, dyes and pharmaceuticals [36,37]. Multianalytical characterization of BC is reported in some recent studies [32,34,38,39].

Due to its physicochemical properties, such as porous structure, large specific surface area and numerous reactive functional groups, BC has a remarkable adsorption capacity which makes it a valid and sustainable alternative to the more expensive activated carbon [40]. The pyrolysis temperature notably influences not only the yield of BC but also its properties and utilization. In general, as the pyrolysis temperature increases from 300 to 800 °C, the calorific value of BC decreases and the porosity increases, while the surface area, which does not change markedly up to 700 °C, increases significantly starting from 800 °C [41]. Low-temperature pyrolysis favors the presence of O-containing functional groups on BC surface and allows a better interaction of the material with solutes of a wider range of hydrophilicity and a better use for agricultural application. Differently, high-temperature pyrolysis favors a higher specific surface area and a greater number of adsorption sites on BC, which allow a better use for environmental purposes [7]. An advantage of this material is that by modulating the values of the process parameters it is possible to obtain a production of BC tailored to the needs of use and the local source of waste biomass [32].

A great effort of recent BC-based research is focused on empowering the adsorption efficiency of this material through specific treatments that can make it competitive with other commonly used adsorbents, such as activated carbons, not only in terms of cost but also in terms of performance. The processes of activation, functionalization and engineering of BC are studied and implemented for this purpose [42]. Postproduction treatments are able to increase the specific surface area, porosity and the content of oxygenated and nitrogenated functional groups of BC. Both chemical and physical activations of BC have been carried out, and in some cases, multiple treatments have also been used. Chemical activation aims to alter functional groups and increase the number of active sites on the surface of the material. Common chemical activating agents for BC are acids, such as H_3_PO_4_ [43], HCl [40] and HF; bases, such as NaOH [40] and KOH [44]; and salts. A very recent technique that seems capable of increasing the adsorbing capacity of this material by up to 10 times consists in doping BC with metals such as Fe also in copresence with other chemicals [45]. Very promising results have been reported by codoping BC with Fe and N_2_ [46]. Another interesting activation technique uses BC and HC for the preparation of magnetic chars by introducing in them metal oxide nanoparticles based on Fe, Ni and Co [47]. Magnetic activation, in addition to improving BC efficiency, has proved effective in facilitating its separation from treated water. Magnetic BC has been used successfully to remove BPA [45] and pharmaceuticals such as 17α-ethinyl estradiol [48] from water.

### 2.2. Hydrochar

The hydrothermal carbonization (HTC) technology is a promising thermochemical process for converting an organic feedstock into a carbon-rich product. During the HTC process, biomass dehydration and decarboxylation generate a marked increase in carbon content, compared to the entering raw biomass. Typical conditions adopted for HTC are elevated temperatures (180–250 °C), autogenic saturated pressure (2–10 MPa) and retention times of some hours [33] (Table 1). The solid product of HTC is a lignin-like material named ‘hydrochar’.

Although the HTC process has been known for nearly a century, only in the last decade has HC aroused growing interest from researchers of the industrial, environmental and agricultural sectors, having proved to be a valuable material. HC exhibits high C content, low atomic O/C and H/C ratios, O-rich functional groups at the surface and a low aromatic structure [32]. Differently from other thermal processes such as pyrolysis which require dry feedstock, HTC has the advantage of using wet biomass with hydrophilic nature and low calorific value without any pretreatment. Like other thermochemical processes, HTC contributes to carbon sequestration since HC recalcitrance allows the fixing of carbon in soil for a long time, thus limiting the greenhouse effect. Since this technology involves little or no pretreatment of biomass, it represents an economically viable solution for producing fuel from wet organic waste such as agricultural waste, food residues, OFMW and sewage sludge. Compared to the raw biomass, HC features low moisture, lower hydrophilicity, more recalcitrance and therefore easier portability and storage. In addition to the type of feedstock, the operating conditions of HTC such as temperature, residence time and pressure are crucial for the final characteristics of HC and its possible applications [11]. In particular, temperature plays a key role in HTC as it allows the thermo-decomposition of the raw biomass. In general, as the process temperature increases, the yield of the solid components decreases, while the liquid and gaseous fractions increase [49]. High temperatures also promote an increase in pH, possibly due to the breakdown of proteins, EC and ash content, while the surface area of HC usually increases up to about 200 °C and decreases at temperatures >200 °C [49]. The type of feedstock is also important because it influences the final composition of HC. Compared to the raw biomass, HC features lower pH, due to the formation of organic acids from lignocellulose breakdown, and much higher EC and ash content. In any case, the parameter that changes the most during HTC is the specific surface area, which increases by tens of times in HC compared to the raw biomass, which is of paramount importance for the sorption efficiency of HC [49]. Due to its physicochemical characteristics, HC is currently mainly used as a solid fuel in conventional combustion processes [9]. However, the good sorption performance of this material suggests the use of HC for the partial replacement of other less eco-friendly and more expensive synthetic adsorbents [50]. Another less explored use of HC is as a soil amendment [51].

Although HC and BC may have similar applications, their physicochemical properties could be dramatically different. Therefore, an extensive characterization is crucial for a preliminary understanding of the sorption capacity of HC. Jian et al. [5] and Gasco et al. [39] determined several chemical and physical properties, such as pH, EC, cation exchange capacity, available P, porosity, elemental composition, contents of total organic carbon, carbonates, metals, moisture, ash, volatile matter, fixed carbon, functional groups and micromorphology of a BC and an HC obtained from the same feedstock and discussed them comparatively. During the HTC process, the original biomass is subjected to a series of chemical reactions such as dehydration, hydrolysis, decarboxylation, polycondensation and aromatization, which drastically change the physicochemical properties of the biomass. A non-negligible difference between HC and BC is the general higher content of cellulose and hemicellulose of HC and, consequently, its higher content of aliphatic carbon and hydrophilic functional groups, which suggests a greater adsorption efficiency of HC towards polar compounds [32]. HC has been shown to be more appropriate than BC for the adsorption of a wider spectrum of organic contaminants [52,53].

In order to enhance the adsorbing performance of HC, researchers have carried out several specific activation treatments during or after the HTC process [54,55]. Common activating agents adopted for HC are alkali metal hydroxides such as NaOH [56] and KOH [57] and single or binary salts [58].

### 2.3. Digestate

The discharge of livestock waste into soil and natural water represents a danger for the environment as it causes sanitation problems, unpleasant odor emission and the eutrophication phenomenon. On the other hand, manure and livestock slurry have contents of organic matter and plant nutrients that can constitute a low-cost supply for improving soil fertility and reducing the use of synthetic fertilizers. Since the organic matter present in such waste has a low C/N ratio and is not humified, and N and P levels can exceed the environmental safety threshold, zootechnical waste must undergo transformation processes before being incorporated into the soil. An economically and environmentally sustainable strategy that is increasingly being adopted around the world is the recycling of this waste, alone or adequately combined with agro-food waste, to produce bioenergy.

The anaerobic digestion (AD) process consists in the biochemical conversion of biowaste operated by bacterial and archaeal populations [59]. The main product of AD is biogas which is a mixture of methane, CO_2_ and small quantities of other gases, while the byproduct consists of a semisolid mixture (about 90–95% moisture) which, after a separation process, usually centrifugation, produces a solid phase and a separate clarified liquid [60].

The separated solid fraction with a moisture content usually lower than 20% and a C content of about 50–55% is called digestate (DG) and is easily transportable and storable. As already discussed for BC and HC, the physicochemical properties of this material greatly depend on the type of biomass entering the digestor, the anaerobic technology adopted and the operating parameters (e.g., retention time, temperature, cosubstrates and working volume) adopted. During AD, easily degradable compounds are readily converted into biogas, while the recalcitrant lignocellulosic fraction remains in the byproduct.

Although this technology dates back several decades, the management of byproducts has always been rather problematic, or at least their proper destination has not been satisfactorily explored so far. In fact, both solid and liquid DGs have been considered mainly wastes to be managed carefully due to the still very high N content and the consequent potential danger for the environment. Up to now, moderate doses of DG have been used for the organic amendment of soil [3]. DG has also been directed to bio-oxidative conversion processes such as composting and vermicomposting [60], used in the preparation of biofilters and biobeds in mixtures with other C-rich substrates [61] or converted thermically to produce BC [41]. However, recent studies have shown that some DG properties, such as the presence of surface reactive functional groups, porosity and a fairly large surface area, can make this material a good candidate for the removal of inorganic and organic contaminants by adsorption [12,62].

## 3. Characterization of Biosorbents

Before using biosorbents for soil and water remediation, it is essential to characterize them. Numerous conventional and advanced techniques are used for this purpose. Choosing the proper methods is crucial to gaining a better understanding of the physical, chemical and physicochemical properties of the material and predicting its sorptive potential. A number of studies report broad, multianalytical characterization of these materials, which includes (i) basic and cheap analyses, such as moisture, ash content, volatile matter and fixed carbon (i.e., proximate analyses), pH, EC and CEC; (ii) more expensive elemental analysis (CHNS-O, i.e., ultimate analysis); and (iii) cutting-edge analyses based on advanced analytical techniques aiming at investigating the surface properties of biosorbents. The advanced analytical techniques include total reflection X-ray fluorescence (TXRF) spectroscopy, scanning electron microscopy (SEM), SEM coupled with energy-dispersive X-ray spectroscopy (SEM-EDX), Brunauer–Emmett–Teller (BET) analysis, Fourier transform infrared (FT-IR) spectroscopy, thermogravimetric (TG) and derivative thermogravimetric (DTG) analyses, nuclear magnetic resonance (NMR) spectroscopy and pyrolysis coupled with gas chromatography and mass spectrometry (Py-GC/MS). Table 2 lists the main analytical techniques used in several characterization studies, while Table 3 shows some results of BC, HC and DG characterization reported in the literature.

The CHNS-O analysis provides the elemental composition of the material and the molar ratios of elements, while TXRF analysis allows quantifying trace and polluting elements in the structure and ash composition [32]. Due to the type of production process, BC generally shows a higher C content than HC and DG (Table 3). Sun et al. [52] measured the organic C content of different BC and HC samples and found values between 53.5% and 65.8% for BC and between 40.2% and 47.5% for HC. A high C content of the material is advantageous for both C storage and adsorption of pollutants. The total C content of BC increases and the O and H contents decrease with increasing pyrolysis temperature, which may be attributed to increased dehydration and decarboxylation of biomass occurring during the process [38]. The atomic H/C and O/C ratios are important parameters for evaluating the degree of carbonization of the material, which strongly depends on the process temperature. High H/C ratios are typical of HC obtained at a temperature around 200 °C and suggest the presence of lignin and cellulose (polar fractions), while low H/C and O/C ratios of BC, which is usually obtained at temperatures ranging between 350 and 800–900 °C, are indicative of high degrees of condensation, aromatization and hydrophobicity [32]. A H/C ratio <0.3 denotes the formation of a highly condensed aromatic structure, while a H/C ratio >0.7 is indicative of an uncondensed structure [38]. The lower C content of HC, compared to BC, is attributable to the lower dehydration and decarboxylation reactions occurring during the HTC process. The DG usually features a total C content between 25 and 41%, on a dry matter basis, with a variability depending on the type of raw biomass processed [6].

The surface micromorphology of both chars and DG can be investigated using the SEM technique, while SEM-EDX analysis allows the evaluation of the composition and the distribution of elements on the surface of the material. Both SEM and SEM-EDX analyses are important for the identification of the type of surface, the size and allocation of pores and the mineral elements present [11,64,69]. The BET analysis is commonly used to measure the specific surface area (m^2^ g^−1^) of the material, and it provides information on the total porosity of the adsorbent, which is very important for the overall sorptive capacity. The surface area of BC has shown positive correlations with the removal of contaminants from soil and water [34]. The BC commonly exhibits diffuse microporosity with pores usually smaller than 1–1.5 nm in diameter, while porosity is less pronounced in HC [32] and much less in DG [12]. The extensive porosity of BC, besides allowing routes for the release of volatile compounds such as H_2_O, CO, CO_2_ and CH_4_ during pyrolysis and providing sites for contaminants, would be an adequate habitat for symbiotic microorganisms once BC is incorporated into soil [10]. The mild temperature used in the HTC process makes HC less or much less porous than BC, which might be attributed to the persistence, during the process, of decomposition products on the surface of HC, which causes pore blockage [52]. Compared to BC, HC has a smaller surface area, although its surface area is sufficiently large to guarantee an appreciable adsorption capacity of molecules with different hydrophobicity [56]. Common features of SEM images of DG are rough surfaces with irregularly shaped ridges, sharp edges, microparticles, channels and cavities mostly smaller than 10 µm. The EDS spectrum of DG usually shows the presence on the surface of elements typical of the raw biomass used in the AD process [12].

The FTIR technique contributes to the understanding of the adsorption mechanisms by providing information on the functional groups of the material and their possible involvement in binding reactions. The surface properties of these materials are dictated by those functional groups that are exposed to the interaction with other surfaces and molecules. FTIR analysis is also used to determine the degree of carbonization and mineralogy of chars. The presence of specific FTIR absorption bands and their relative intensity are indicative of ionizable functional groups (hydroxyl, carboxyl, phenolic, carbonyl, amino and sulfhydryl) capable of interacting with ionizable compounds, as well as the presence of aromatic structure in the material. The FTIR spectra of BC, HC and DG are quite different, denoting different structural properties of these materials. Typical peaks observed in FTIR spectra of BC can be assigned to O-H stretching of inter- and intramolecular hydrogen bonds and N-H stretching (3420–3440 cm^−^^1^), methyl C-H stretching (~2930 cm^−^^1^), methylene C-H stretching (~2860 cm^−1^) and aromatic carbonyl/carboxyl C=O (~1700 cm^−^^1^) and aromatic C=C (~1600 cm^−1^) stretching vibrations [70]. The absorption bands at ~1400 cm^−1^ are assigned to phenolic O-H and C-O bonds which promote the immobilization of pollutants through complexation, while the bands at ~1110 and ~870 cm^−1^ can be assigned to aromatic C-H deformation [71]. In addition to the peak at 3420–3440 cm^−1^ (O-H and N-H stretching), FTIR spectra of HC usually feature peaks at ~2920 and 2850 cm^−1^ (asymmetric and symmetric stretching of aliphatic C-H, respectively); ~1620 cm^−1^ (aromatic C=C stretching); at 1310, 1160, 1110 and 1030 cm^−1^ (stretching vibrations of C-H in hydroxyl, ether or ester and bending vibrations of O-H in cellulose and hemicellulose); 670 (out-of-plane bending vibration of C-H); and 580 cm^−1^ (bending vibration of O-H) [32]. The main features of FTIR spectra of DG are the presence of a wide absorption band at ~3300–3400 cm^−1^ (O-H vibration of carboxylic and alcoholic groups and N-H stretching) and peaks at ~2920 and ~2850 cm^−1^ (aliphatic C-H stretching), ~1630–1600 cm^−1^ (various vibrations, including aromatic C=C stretching), 1385 cm^−1^ (various vibrations, including O-H deformation and C-O stretching of phenolic groups, COO^-^ asymmetric stretching) and ~1040 cm^−1^ (C-O stretching of polysaccharide-like substances and Si-O vibration of silicate impurities) [12].

The TG and DTG techniques allow the evaluation of the structural stability of the adsorbent and the changes of physical and chemical properties occurring during heating, which can be achieved by monitoring the weight loss pattern caused by the heating rate under controlled atmospheric conditions (air, He or N_2_). The TG analysis is important for processed materials as it highlights and differentiates their thermal decomposition. The first phase of mass loss is due to the loss of moisture (dehydration) and occurs between 50 and 150 °C, the second one is measurable between 150–200 and 360 °C and is mainly related to the thermal degradation (volatilization and decomposition) of hemicelluloses and cellulose and the third one is attributed to the degradation of lignin and occurs between 360 and 600 °C. Chars from lignocellulosic biomass feature more markedly the three stages of decomposition compared to chars from mixed plant and animal biomass [32]. The TG and DTG analyses were used by Missaoui et al. [65] in studying changes of physical and chemical properties of an HC from olive pomace during heating. The TG analysis of a DG from swine manure clearly showed three distinct stages corresponding to (i) loss of moisture and volatile compounds (up to 200 °C), (ii) decomposition of lignocellulosic residues still present after AD (from 200 to 800 °C) and (iii) decomposition of inorganic minerals such as calcite and calcium phosphates (from 800 to 1000 °C) [41].

NMR is another technique commonly used to investigate the structural composition of biosorbents. This technique provides details on the type of functional groups of the material and the proportion between aliphatic and aromatic fractions. Moreover, it allows the evaluation of the degree of carbonization of chars and their stability. Solid-state ^13^C NMR spectra reveal the main types of functional groups (e.g., aliphatic, aromatic, phenolic, methoxyl) of the material and usually show significant differences between BC and HC. Usually, ^13^C NMR spectra of BC are dominated by a huge peak at ~120–130 ppm that is characteristic of aromatic C [72]. The contribution of aryl C (108–148 ppm) in BC is greater than that in HC, and the contribution of alkyl C (0–45 ppm) in BC is lower than that in HC, denoting a lower degree of carbonization of HC. The NMR spectra of HC are more structured than those of BC and show significant signals of carbohydrates (63–108 ppm) and carboxyl C (165–187 ppm) [52]. Fierro and coworkers [68] used ^1^H NMR to characterize DG obtained from swine manure and found high-intensity peaks in the aliphatic region, especially at 1.22 ppm ascribed to CH_2_- groups, 1.7 ppm ascribed to unsaturated compounds and 7.2 ppm associated with the coupling effect of methylene protons in benzene rings.

Finally, the Py-GC/MS technique is used to investigate the key marker compounds present within biosorbents. The Py-GC/MS pyrograms allow the identification of a list of compounds, mainly aromatic hydrocarbons and volatile organic compounds, present in the material based on release time and m/z value. The key compounds of the material are identified by the intensity of the peaks present in the pyrogram. Depending on the source materials used, the common components of chars include benzene, ethylbenzene, naphthalene, xylene and styrene [32].

## 4. Environmental Estrogens

### 4.1. Phenolic Estrogens

Some alkylphenols are industrial products and byproducts that have attracted the attention of researchers due to their estrogenic activity and their diffusion in the environment at doses generally higher than those of other EDCs of different chemical nature, such as natural and synthetic estradiols [26]. Due to their toxicity and persistence, PhEEs are of great concern as they have detrimental effects on human health and environmental safety [19]. The role of these compounds has been demonstrated in the context of several pathologies in males and females, such as reproductive system and thyroid dysfunction, immunotoxicity, development of breast and prostate cancer, impaired metabolism and obesity, diabetes and cardiovascular dysfunctions [15,21].

This group of estrogens includes several compounds that are constituents of a wide range of common consumer products and products and byproducts of industrial manufacturing processes, such as surfactants, detergents, pesticides, paints, dyes and pharmaceuticals. They are continuously released into natural water bodies with the discharge of effluents from sewage treatment plants [73]. Primary sources of PhEEs in natural and cultivated soil include the application of sewage sludge, irrigation with wastewater and the discharge of landfill leachate [74].

Among PhEEs, (2,2-bis(4-hydroxyphenyl) propane (BPA), OP and nonylphenol (NP) have similar structural features and behavior in the environment. Some properties of these compounds are shown in Table 4. BPA, OP and NP are widely adopted in industrial processes for the preparation of items manufactured for daily use, such as electrical and electronic parts, medical equipment (e.g., dental prostheses and sealants), flame retardants, adhesives, paints and food and beverage packaging [73].

Currently, BPA is one of the chemicals produced in the highest quantities worldwide, with approximately 8 million tons produced per year [49], and much more is expected to be produced in the future. BPA is the building block for the preparation of epoxy resins and polycarbonate plastics and is used as a stabilizer for plastics such as polyvinyl chloride (PVC). BPA is widely present in everyday items, such as plastic bottles, paints, food cans, plastic toys, electronic equipment, paper and cardboard products, furniture, building materials, water pipes, footwear and leather products [76]. Due to its endocrine-disrupting activity, BPA has been banned in baby bottles in the European Union since June 2011 [25].

BPA reaches the environment from both preconsumer and postconsumer sources. The preconsumer sources derive from the industrial production of BPA-containing plastics and epoxy resins and their discharge via industrial wastewater. Postconsumer sources include effluent discharge from municipal WWTPs, landfill leachates and degradation of plastics in the environment [74]. BPA is a moderately water-soluble compound (Table 4) and is believed to possess moderate bioaccumulation [77]. The low vapor pressure and consequently the low volatility of BPA is the reason for its persistence mainly in the hydrosphere. A long-term BPA monitoring study conducted in North American and European fresh and marine surface waters reports 95th percentile concentrations of 0.30 μg L^−^^1^ for both geographical areas and 0.024 μg L^−1^ and 0.15 μg L^−1^ in marine water, respectively [22]. In WWTP effluents, BPA concentration ranges from undetectable or a few micrograms per liter to 370 μg L^−1^ [21]. This molecule is ubiquitous in sewage sludges and biosolids, with concentrations on dry weight ranging from 10 to 100,000 μg kg^−1^, and can also be much more concentrated in sludge from WWTPs [21]. The BPA concentration in soil varies over several orders of magnitude, i.e., from less than 0.01 to 1000 μg kg^−1^ depending on the amount and type of effluent or waste received, while more than 20,000 μg kg^−1^ of BPA was found in downstream sediments of heavily polluted urban areas [21].

Other well-known molecules with estrogenic properties are the two alkylphenols OP and NP which are the precursors of the nonionic surfactants octylphenol polyethoxylates (OPEOs) and nonylphenol polyethoxylates (NPEOs) that are used in the formulation of paints, detergents, personal care products, pesticides, lubricants, emulsifiers and so on. As a result of the microbial degradation of OPEOs and NPEOs, OP and NP are released into the environment where they exert a greater toxicity than their parent compounds and can cause estrogenic effects on aquatic fauna and humans [73]. OP has higher estrogenic activity than NP, and both can bind to estrogen receptors of animals and cause disorders of reproductive functions [78]. OP and NP are widely present in natural waters, especially river water, due to the discharge of domestic and industrial wastewater and rain runoff. The concentrations of OP and NP in natural waters generally range between 23 and 255 ng L^−1^ [19,79] but can also reach some micrograms per liter [80]. OP and NP are also present in WWTP effluents and can be released in the environment where they persist for a long time due to their significant recalcitrance to biodegradation [81].

### 4.2. Steroidal Estrogens

The StEEs belong to the class of EDCs and include natural compounds involved in the maintenance of female functions, such as estrone (E1), 17β-estradiol (17β-E2), 17α-estradiol (17α-E2) and estriol (E3), as well as the synthetic estrogen 17α-ethinyl estradiol (EE2). E1, E2 and E3 are produced by all vertebrates and some insects, while EE2 is widely adopted as a contraceptive pharmaceutical, as a menopause-related drug and in the therapy of human diseases such as breast and prostate cancer and osteoporosis [82]. Some properties of these compounds are reported in Table 4. The estrogenic potency of StEEs, in particular E2 which is the strongest, is significantly greater than that of PhEEs, but they are generally detected less frequently and at lower levels in aquatic environments [82]. The overall estrogenic potential of an EE is usually expressed quantitatively via the estradiol equivalent (EEQ) parameter since the reference for estrogenicity for all EEs is the compound 17β-estradiol [82].

The StEEs are widespread water contaminants and are of great concern due to their potential hazard to the health of aquatic animals and humans. It is estimated that ~30 tons/year of natural StEEs are released into the environment by the global human population, along with ~0.7 tons/year of synthetic EE2, while in the US and the EU, the overall cattle industry generates ~83 tons/year of estrogens [82]. The StEEs are released as liquid and solid animal and human excreta in municipal and rural waterways, especially in densely populated areas, and accumulate in wastewaters. The direct animal manure application in agricultural land can contribute to the release of steroid estrogens into soil with potential subsequent transfer to aquatic systems. The use of animal manure to produce biogas from AD can determine the presence of these compounds in both solid and liquid digestates which are often applied to soil as organic amendments [83]. Other sources of StEEs are pharmaceutical and hospital wastes. The StEEs are typically excreted into the environment in conjugated form but are rapidly converted to unconjugated molecules [82]. The EE2 molecule containing an ethynyl group is more stable and shows greater resistance to oxidative reactions than other estrogens [82]. Consequently, EE2 has a longer persistence in the environment than natural estrogens and therefore is even more dangerous. Furthermore, EE2 has an estrogenic potency estimated 1.25 times higher than that of E2, which is the most potent natural estrogen [84].

The nonadsorptive and adsorptive methods commonly used for wastewater treatment, such as activated sludge and advanced oxidation processes (AOPs), are quite expensive, generate toxic byproducts during treatments and are unable to completely remove these compounds [28]. The use of not completely depolluted effluents for crop irrigation releases these compounds into soil and natural waters after leaching and runoff. Due to the lack of effective removal treatments, StEEs have been detected in natural waters after the discharge of municipal wastewater effluents and landfill leachate from waste disposal [84]. A survey of StEE levels in effluents from sewage and WWTPs collected at numerous European sites reported that E1, 17α-E2, 17β-E2 and EE2 were present at concentration ranges of 12–197 ng L^−1^, 6–13 ng L^−1^, 6–43 ng L^−1^ and 1–6 ng L^−^^1^, respectively [85]. An extensive monitoring of the presence of EE2 in the incoming and outgoing effluents of WWTPs of 282 municipalities across 29 countries has detected EE2 concentrations ranging from 0 to 7890 ng L^−1^ in influents and from 0 to 470 ng L^−1^ in effluents originating from the activated sludge processes [84]. In a Chinese river, Wang et al. [79] found levels of E1, E2, EE2 and E3 of up to 56, 24, 31, and 5 ng L^−1^. StEEs have also been detected in wildlife, mostly fish, where they bioaccumulate and reach very high concentrations [82].

Even at ppt levels, these compounds can alter hormonal mechanisms in animals, producing harmful effects such as feminization of aquatic fauna, infertility and cancer [86]. For these reasons, StEEs have increasingly drawn the attention of the community and regulatory authorities. Data from the global monitoring of StEEs are of increasing concern, and therefore more robust environmental assessment and management programs are needed, especially in more urbanized areas.

## 5. Technologies for the Removal of Estrogens

**Figure 1:**
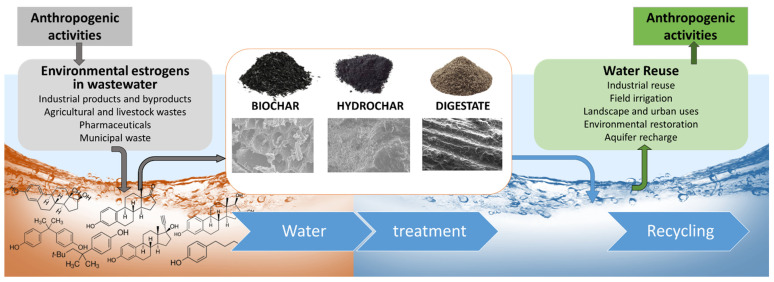
Water recycling.

Water scarcity caused by population growth, economic development, current styles of consumption and climate change has become an urgent emergency. The growing demand for water from the industrial sector and, especially, from agriculture, which is the main water user (~70% of the global water demand), requires great attention to water resources [87]. In the next years, water demand will grow, particularly in countries with developing or emerging economies [29]. Pure water is precious for living beings and healthy agricultural productions, and its availability is not unlimited. Another important issue is the expected relevant increase in municipal wastewater due to rapid urbanization. Furthermore, the ever-increasing intensive anthropic activities continuously deteriorate the quality of the environment, and therefore timely and sustainable solutions that rely on the use of low-cost natural resources are required. These problems are emerging more and more widely in both industrialized and developing areas. The elimination of EEs represents a serious economic and environmental challenge. Finding sustainable solutions for wastewater reclamation and reuse will reduce the risk of environmental contamination and provide an additional supply of water for agricultural, industrial and civil uses (Figure 1). A schematic overview of the current technologies available for the removal of EEs from water and soil is shown in Table 5.

Nowadays, the removal of micropollutants such as EEs from the environment is not adequately carried out and perhaps not even sufficiently explored. Therefore, the detection of these compounds in ecosystems continues to be reported [21,22]. The processes conducted in the WWTPs aim mainly to remove the organic load and nutrients from wastewater. Conventional technologies studied and developed in recent years for the treatment of wastewaters and other environmental matrices are essentially based on chemical, physicochemical and microbiological processes. Depending on the nature of the contaminants, the applied processes can be classified into three categories: containment immobilization, separation and destruction [26,89]. Current treatments are based on flocculation, precipitation–filtration, adsorption on activated carbon, membrane filtration (micro-, ultra- and nanofiltration, reverse osmosis), advanced chemical and electrochemical oxidation and photocatalytic degradation [27,29]. Biological treatments are essentially based on the use of activated sludge, which is a mixture of microorganisms and suspended solids. Unfortunately, these techniques are not always resolutive, can generate hazardous byproducts and are extremely expensive.

Despite the numerous studies carried out on this matter, to date, there is no specific process that can be applied to completely remove EEs from wastewater [84], and tertiary-stage treatments usually include chlorine disinfection. Furthermore, multicontamination is the norm in water and wastewaters. The coexistence of many EEs in water is very likely due to intense agricultural and industrial activities and large volumes of wastewater discharged. Furthermore, the co-occurrence of pollutants exerts highly dangerous synergistic adverse effects on animal health [95]. Gao et al. [28] have recently reviewed scientific information on the available eco-sustainable methods for the removal of EEs in wastewaters with a focus on photocatalysis and biodegradation.

An affordable and ecological solution to avoid, or at least limit, environmental pollution from EEs might be to combine emerging technologies and new biobased materials with existing technologies to improve their performance or overcome limitations such as high costs [13]. Based on this scenario, new sustainability-oriented strategies have been recently proposed to remove EEs from water and solid environmental matrices at affordable costs, including new biobased technologies for full-scale implementation. Biobased technologies appear to be an interesting economic alternative and easily meet people’s consensus for their nature-friendly approach. Examples of biobased remediation technologies used for the removal of PhEEs and StEEs from environmental matrices are phytoremediation, which uses algae [96] and higher plants [94] to absorb and transform organic pollutants, and bioremediation, which uses microorganisms [97,98].

Adsorption techniques consist in the retention of organic pollutants present in solution on the surface of a solid adsorbent. Industrial adsorbents, such as activated carbon, carbonaceous resins and modified zeolites, have been widely used for removing single or multiple contaminants. However, the high costs of these synthetic adsorbents limit their use. Innovative solutions explored in recent years for wastewater remediation are based on the use of adsorbents deriving from both raw biowaste [99] and processed biowaste of bioenergy technologies [49,100]. In a medium-term perspective, and especially in low-income countries, the latter category of materials that are effectively waste from waste treatment could be valid competitors of much more expensive synthetic materials and replace them at least in part.

## 6. Sorptive Removal of Estrogens by Biosorbents

### 6.1. Adsorption Kinetics and Equilibrium Isotherms: Type of Interaction and Data Modeling

The characterization of biosorbents, in addition to providing useful information on their structural and functional properties, is useful for understanding the potential binding mechanisms with contaminants. The adsorption process can be generally classified as either physisorption (physical adsorption) or chemisorption (chemical adsorption). Physisorption is generally more common than chemisorption and occurs through weak intermolecular forces, such as van der Waals forces and hydrogen bonding, which do not involve a significant change in the electronic state of interacting species. Chemisorption includes valence forces, such as covalent or ionic bonds, between the binding sites of the adsorbent and solute. The main differences between physisorption and chemisorption relate to the enthalpy of adsorption, reversibility and layered arrangement (one or more layers) of the solute on the adsorbent. Physisorption is a low-enthalpy and reversible process that usually involves multiple layers of solute, while chemisorption is a high-enthalpy and irreversible adsorption that generally involves one monolayer of solute on the adsorbent. Generally, the adsorption of EEs on biosorbents occurs through more than one mechanism and is strongly dependent on the pH of the medium, which influences the electric state of the EE and the adsorbent. Furthermore, the type of prevailing bonds depends on the extent and type of functionalities of the material, which, in turn, depend on the parameters of the production process [100]. In general, low pyrolysis temperatures favor the formation of O-containing groups on BC, while high temperatures favor carbonization and aromatization reactions which make BC more suitable for adsorption of hydrophobic compounds [7]. BC interaction with PhEEs, such as BPA, and StEEs, such as EE2, has been mainly attributed to π–π electron donor–acceptor binding, with the solute acting as π-donor, along with H bonds between phenols and polar sites of BC [52]. The mild temperature adopted in the HTC process is responsible for the high number of O-containing functional groups and the formation of H bonds with EEs, with both interacting species (HC and EE) generally acting either as acceptors or donors [52]. Other mechanisms involved in the EE–HC interaction are π–π electron acceptor–donor bond, electrostatic interaction and hydrophobic interaction [49,53]. The molecular size of EEs is also an important property for their adsorption on porous chars, and the pore-filling mechanism can play an important role in this process [52]. Very little information on EE–DG interaction is available in the literature. Spectroscopic analyses of DG show the presence of carboxylic, alcoholic and phenolic –OH; amino groups; and aliphatic and aromatic C, suggesting the formation of the same bonds formed by BC and HC [12]. Further studies are needed to investigate the mechanisms of EE–DG interaction.

The adsorptive capacity of a biosorbent for an EE is quantified by studying adsorption kinetics and equilibrium isotherms. Modeling of experimental sorption data is a widely accepted approach to evaluate and compare the adsorption performance of different materials for a given molecule and the affinity of different molecules for the same material.

Kinetics data allow the estimation of the rate of adsorption and the equilibration time and are usually interpreted using various kinetic equations. Based on the type of model that provides the best correlation of experimental data, information can be obtained on the sorption mechanisms, i.e., physisorption or chemisorption. Theoretical kinetic models commonly used to interpret experimental data of EEs on biosorbents are the pseudo-first-order (PFO), the pseudo-second-order (PSO), Elovich’s and intraparticle diffusion (IPD) models [43,52,101,102]. The equations and parameters of the four sorption kinetics models are shown in Table 6.

The PFO rate equation of Lagergren [103] is based on the adsorbent capacity and considers that a linear relationship occurs between the speed of occupation of the active sites and the number of sites available on the adsorbent [104]. The PSO kinetic model is based on the sorption at equilibrium and provides information on the type of interaction between the sorbent and the sorbate, such as valence bond and electron exchange [105]. According to the PSO model, the rate-limiting step of adsorption may be the chemisorption of the solute onto the adsorbent through covalent bonds. The Elovich model allows the evaluation of the existence of chemisorption and assumes that the adsorption rate of the solute decreases exponentially as the amount of adsorbed solute increases [106,107]. The IPD model is applied to know the limiting step of adsorption and assumes that the only interaction between solute and adsorbent is the internal diffusion [108]. Numerous studies demonstrated that the PSO kinetic model is the best fit for the adsorption of most EEs on BC [43,46,90] and HC [56,57,58].

Adsorption isotherms are performed to quantify the distribution of a solute between the adsorbent and the solution at equilibrium. The experimental equilibrium data are generally fitted into a sorption model to calculate the adsorption constants (Table 6). These models can also be useful for understanding the sorption mechanisms occurring at the solid–liquid interface. Two commonly used theoretical sorption isotherm models are the Freundlich and the Langmuir equations. The empirical nonlinear Freundlich model [109] fits well when the solute is adsorbed on heterogeneous substrates, while the Langmuir isotherm [110] is more appropriate for materials having a homogeneous surface and when the solute is adsorbed as a monolayer without interaction between solute molecules. The Freundlich model was the best interpretation of equilibrium sorption data of various EEs on BC [52,53,101], HC [52,58] and DG [12]. Differently, other works demonstrated that the Langmuir model was the most appropriate fit for EEs on BC [43,46,90] and HC [57,102].

**Table 6:** Models of adsorption kinetics and equilibrium isotherm for environmental estrogens (EEs) on biosorbents.

Ref.	Model	Equation	Parameters
Adsorption Kinetics Models			
[103]	Pseudo-first order	*q_t_* = *q_e_* (1 − *exp^−k^*^1^^*t*^)	*q_e_* and *q_t_* are the amount of solute adsorbed per mass unit of sorbent (mg g^−1^) at equilibrium and at time *t*, respectively; *k*_1_ (h^−1^) and *k*_2_ (kg mg^−1^ h^−1^) are the rate constants of sorption
[105]	Pseudo-second order	$q_{t}=\frac{q_{e}^{2}k_{2}t}{1+k_{2}q_{e}t}$	
[106]	Elovich	$\frac{dq}{dt}=a$ *e^−αq^*	*q* is the amount of sorbate adsorbed at time *t*, *a* is the desorption constant and *α* is the initial adsorption rate
[108]	Intraparticle diffusion	*qt* = *k_id_* *t*^1/2^ + *C*	*q_t_* is the amount of solute adsorbed per mass unit of sorbent (mg g^−1^) at time *t*, *k_id_* (mg g^−1^ min^−1/2^) is the particle diffusion rate constant and *C* (mg g^−1^) is the intercept
Adsorption Isotherm Models			
[109]	Freundlich	*q_e_ = K_F_ C_e_* ^1/*n*^	*q_e_* (mg g^−1^) is the amount of solute adsorbed per unit of substrate; *C_e_* (mg mL^−1^) is the equilibrium concentration of the sorbate in solution. 1/*n* indicates the degree of nonlinearity between solution concentration and amount adsorbed, while the reciprocal *n* is the sorption intensity, *K_F_* is the Freundlich adsorption constant, *b* (mg g^−1^) is the maximum monolayer adsorption, *K_L_* (mL mg^−1^) is the Langmuir constant which expresses the energy of adsorption and *Kd* (mL g^−1^) is the distribution coefficient
[110]	Langmuir	*q_e_* = (*K_L_C_e_b*)/(1 + *K_L_C_e_*)	
[111]	Henry	*q_e_ = Kd C_e_*	
[112]	Temkin	*qe* = *B ln*(*A_T_ C_e_*)	*q_e_* (mg g^−1^) is the amount of solute adsorbed per unit of substrate, *C_e_* (mg mL^−1^) is the equilibrium concentration of sorbate in solution, *A_T_* is the Temkin isotherm equilibrium binding constant (mL mg^−1^) and *B* (J mol^−1^) expresses the enthalpy of adsorption. *B* = *RT/b_T_*, where *b_T_* is a constant related to the heat of adsorption, *T* is the absolute temperature (K) and *R* is the universal gas constant (8.314 J mol^−1^ K^−1^)
[113]	Dubinin–Radushkevich	*qe* = (*q_s_*) *exp* (−*k_ad_^2^*)	*q_s_* (mg g^−1^) is the maximum sorbate adsorption; *k_ad_* is the Dubinin–Radushkevich constant (mol^2^/kJ^2^). *k_ad_* = A/E, where E (kJ/mol) is the energy associated

Less common models used to fit equilibrium isotherm data are the Henry, the Temkin and the Dubinin–Radushkevich models (Table 6). The Henry equation assumes a constant proportion between the concentration of the sorbate in solution and that on the adsorbent and allows the calculation of the distribution coefficient, Kd (mL g^−1^), from the slope [111]. The Temkin isotherm is based on the interaction among adsorbate molecules on the adsorbent surface and predicts a logarithmic reduction in adsorptive sites and energy [112]. Finally, the Dubinin–Radushkevich model is widely used to describe the adsorption of molecules in microporous chars and assumes a different energy released in the physical and chemical adsorption [113]. Furthermore, to better compare the capacity of different biosorbents to retain a given EE, the partition coefficient *K_OC_*, i.e., the quantity of adsorbed compound per unit of organic C of the adsorbent, is generally calculated according to the following: *K_OC_* = (*K_d_*/(*% organic C*)) × 100.

An important aspect of biosorption is the capacity of the material to retain/release the compound when external conditions change. Therefore, an adsorption study is usually complemented by a desorption study. The desorption data, i.e., the amounts of a compound that remain adsorbed at each desorption step and the corresponding equilibrium concentrations, are generally interpreted by the Freundlich equation to calculate the desorption parameters *K_Fdes_* and 1/*n_des_*. The hysteresis coefficient, H, is calculated from the ratio H = (1/*n_des_*)/(1/*n_ads_*).

### 6.2. Removal from Water and Soil

#### 6.2.1. Biochar

Searching on the Scopus database for ‘biochar’ in the article title, abstract and keywords (AT&A&K) resulted in 21187 BC-based documents (articles, conference papers, reviews, book chapters, books and so on) listed since 2000 (queried on 10 January 2022), of which over 78% were published in the last five years. Most of these studies were carried out in China (45%), the United States (15%), India (6%) and Australia (6%), and the remaining ones were carried out in 139 other countries. A large number of these studies focused on the characterization, properties and applications of BC; searching for the combination ‘biochar’ and ‘adsorption’ or ‘sorption’ or ‘removal’ in the AT&A&K resulted in the identification of 8182 documents in 27 different subject areas, the main one being environmental science (69.2%). Upon limiting this latter search to the seven EEs considered in this review (BPA, OP, NP. E1, E2, E3 and EE2), about 140 documents were identified; as expected, most of them concern BPA (74 documents).

In all works, BC showed an excellent sorptive efficiency which suggests its valid use for water treatment and soil remediation. The addition of BC to soil produces multiple benefits, including the increase in recalcitrant organic matter, the retention of water and plant nutrients and the immobilization of contaminants. The last function is very important because it avoids the entry of contaminants, especially the less hydrophobic ones, into natural waters and into the food chain after plant absorption and accumulation in edible parts [51,114,115].

BC has been used as an adsorbent both unmodified and after a series of chemical and physical postproduction treatments (activation, functionalization, magnetization, engineering). Activation can empower BC performance by increasing its specific surface area, porosity, content of oxygenated functional groups and ability to retain pollutants with a wider range of hydrophobicity.

The mechanisms by which BC interacts with EEs depend on the type and number of chemical functional groups on its surface, which, in turn, vary according to its production conditions, above all pyrolysis or gasification temperature. In general, lower temperatures cause the formation of numerous O-containing groups on BC, while higher temperatures favor carbonization and aromatization reactions which make the material more suitable for retaining hydrophobic compounds [7].

The high aromaticity of BC can explain the strong retention of PhEEs, such as BPA, through the formation of π–π electron donor–acceptor bonds with BPA acting as π-donor (–OH substituted aromatic compounds), along with the interaction of phenols with polar aromatic cores of BC [52]. The occurrence of H bonding between BC and polar functional groups of PhEEs is also reported [40]. The smaller molecular size of PhEEs, compared to StEEs, would favor their access to sorption sites of BC. In the StEE–BC interaction, π–π electron donor–acceptor binding is considered to be the dominant mechanism, although H bonding and electrostatic attraction are also common depending on the surface charge of BC and the possible ionization of StEEs [100].

When BC interacts with mixtures of EEs, a positive correlation between the adsorption constants, or organic C-normalized adsorption constants, and the Kow values of EEs is commonly observed [53]. However, this correlation is often not statistically significant, which would suggest that the interaction may be governed by other important factors, such as the molecular size of the EE or the arrangement of functional groups on BC. Furthermore, pore filling is an important mechanism in the adsorption of hydrophobic compounds on BC, which is confirmed by the inverse relationship between the adsorption capacity of the char and the molecular diameter of the EE [52].

A selection of studies on the adsorption of EEs on BC shows that the PSO model is the preferred fit for kinetics data (Table 7), indicating the occurrence of chemisorption, via π–π electron donor–acceptor bonds and electrostatic interactions, between solute and sorbent.

In equilibrium isotherm studies, the experimental data follow different models depending on the type of BC and the EE considered (Table 7). In some studies, the Freundlich equation is the best fit for BPA [52,116,117], OP and NP [118], E1 [53,91], E2 [101] and EE2 [50,119] adsorption on BC. The Freundlich-type model is appropriate for adsorbents featuring heterogeneous surface and when solute molecules interact with each other and form a multilayer coverage on the adsorbent. In other studies, data of BPA [40,43,45,46], OP [120] and any StEE [43] seem better correlated with the Langmuir equation. This model is appropriate when the solute molecules form a monolayer on the homogeneous surface of the adsorbent and do not interact with each other. Furthermore, the activation of BC does not appear to change the preferred isotherm model for BPA [45,46] and E2 [44], compared to the unmodified material.

Freundlich adsorption constants (K_F_) and Langmuir maximum adsorption capacity (Q_max_) express the amount of solute adsorbed on the sorbent at equilibrium. Loffredo and Taskin [120] found Langmuir-type and Freundlich-type isotherms for sorption of OP and E2, respectively, on BC and PSO kinetic model for both EEs, which indicates chemisorption as the main interaction mechanism. Very high sorption capacity of BC for StEEs is documented in the scientific literature [44,53]. Ahmed et al. [43], investigating the sorption of BPA, E1, E2, E3 and EE2 on functionalized BC, found that the Langmuir equation and the PSO kinetic model were the preferred fit for all compounds and reported H bonds and π–π electron donor–acceptor binding as the main interaction mechanisms.

**Table 7:** Removal of different environmental estrogens (EEs) from water using biochar.

EE	Feedstock	Process T (°C)	Residence Time (h)	Activation	Kinetics Model	Isotherm Model	Adsorption Capacity (mg g^−1^) *	Ref.
BPA	Wheat straw	400	2–7	-	n. e.	F	9.33	[52]
BPA	Poultry litter	400	2–7	-	n. e.	F	57.54	[52]
BPA	Pine chips	800	2	-	n. e.	F	9.22	[116]
BPA	Eucalyptus wood	600	2	oH_3_PO_4_	PSO	L	47.65	[43]
BPA	Alfalfa	650	2	-	PSO	F	24.30	[117]
BPA	Grapefruit peel	400	2	-	PSO	L	123.83	[45]
BPA	Grapefruit peel	400	2	γ-Fe_2_O_3_	PSO	L	342.47	[45]
BPA	Wheat straw	550, 700	0.75	CO_2_	n. e.	L	17.5, 14.2	[121]
BPA	Wheat straw	700	2	NaOH	PSO	L	71.42	[40]
BPA	Sawdust	800	1	-	PSO	L	5.08	[46]
BPA	Sawdust	Various (from 500 to 900)	1	Fe and N_2_	PSO	L	From 0.9 to 54.0	[46]
OP	Red spruce wood pellet	550	3	-	PSO	L	1.79	[120]
OP	Sawdust	450, 650, 850	1	-	n. e.	F	0.56, 1.05, 0.63	[118]
NP	Sawdust	450, 650, 850	1	-	n. e.	F	1.50, 2.07, 1.07	[118]
E1	Swine solids	Various (from 250 to 600)	2	-	n. e.	F	From 1.71 to 665.18	[53]
E1	Poultry litter	Various (from 250 to 600)	2	-	n. e.	F	From 4.77 to 460.47	[53]
E1	Eucalyptus wood	600	2	oH_3_PO_4_	PSO	L	75.88	[43]
E1	Lychee fruits	650	2	-	PSO	F	0.65	[91]
E2	Pine chips	800	2	-	n. e.	F	30.20	[116]
E2	Red spruce wood	550	3	-	PSO	F	3.385	[120]
E2	Rice straw	Various (from 400 to 600)	2	-	PSO	F	From 6.86 to 13.95	[101]
E2	Eucalyptus wood	600	2	oH_3_PO_4_	PSO	L	51.26	[43]
E2	Lotus seedpod	650	2	-	PSO	L	135.74	[44]
E2	Lotus seedpod	650	2	KOH	PSO	L	150.10	[44]
E3	Eucalyptus wood	400	2	oH_3_PO_4_	PSO	L	42.17	[43]
EE2	Wheat straw	400	2–7	-	n. e.	F	29.51	[52]
EE2	Poultry litter	400	2–7	-	n. e.	F	8.32	[52]
EE2	Peanut shell	550	2	-	n. e.	F	80.44	[119]
EE2	Eucalyptus wood	400	2	oH_3_PO_4_	PSO	L	51.16	[43]

* Data refer to K_F_ or Q_max_ based on preferred Freundlich or Langmuir isotherm, respectively, at temperature of 25 ± 2 °C; K_F_: Freundlich constant; L: Langmuir isotherm; F: Freundlich isotherm; PFO: pseudo-first order; PSO: pseudo-second order; n. e.: not evaluated; -: no treatment.

Due to its high sorption capacity, BC can also play an important role in soil remediation. The addition of BC to soil significantly increases its ability to retain EEs, thus limiting the danger of their leaching to the deeper soil layers and transport to natural waters. This important BC action has been demonstrated in soils naturally or artificially contaminated with BPA [90,92,93,94,122], OP [94], E1 and E2 [123] and EE2 [92,93]. Furthermore, the retention of EEs by BC in soil is strongly influenced by the level and type of natural organic matter, in particular the humic fraction, and by dissolved organic matter [122]. Recently, BC and other carbon-rich materials have been used to remove BPA and OP from soil in a new strategy that does not involve the incorporation of BC into soil. This approach consists in a preliminary adsorption of contaminants on BC which is subsequently removed from the soil and exposed to fungal activity for the degradation of contaminants [37]. Using this method, in just 2 days of soil–BC contact, up to 80% and 62% of OP and BPA, respectively, can be permanently removed from soil, and a large part of the contaminants (83% and 75% of OP and BPA, respectively) is successively eliminated through mycodegradation [37].

#### 6.2.2. Hydrochar

The HTC process produces a carbonaceous material that exhibits good performance as an adsorbent of organic and inorganic contaminants in aqueous solutions. HTC is a relatively recent and less widespread technique compared to other thermochemical processes such as pyrolysis, gasification and combustion; therefore, in the scientific literature, the number of HC-based studies is much more limited than that of those concerning BC. The number of studies on HC surveyed by Scopus database (AT&A&K) is 1856, and these studies were published in 2009 or later (queried on 8 January 2022). Of all these studies, a very large percentage (82%) was published in the last 5 years, and 50% were published in the last 2 years. The majority of HC-based studies focus on the HTC process and on HC characterization and applications in different sectors. Combining the words ‘hydrochar’ and ‘adsorption’ or ‘removal’ and searching in AT&A&K resulted in 558 articles, of which only about 15 are related to PhEEs and StEEs. Therefore, although HC as an adsorbent has significantly attracted the attention of scientists, its potential for EE retention/removal is still very little explored.

The capacity of HC to retain pollutants strongly depends on the type of raw biomass used and on HTC parameters (temperature, residence time, pressure, raw material/liquid ratio). In general, the sorption efficiency of HC is lower than that of BC, which is due to a smaller specific surface area and lower porosity that provide fewer active sorption sites for pollutants. However, due to the presence of different types of functional groups on HC, this material is effective for the removal of both polar and nonpolar contaminants [52,53,102].

The mechanisms by which EEs are bound by HC are different and depend on the EE considered and the pH of the medium. They can be listed as π–π electron acceptor–donor bond, H bond, electrostatic interaction and hydrophobic interaction [49,53]. Due to its high content of oxygenated functional groups, HC can form H bonds by acting either as an acceptor or a donor. In addition, EEs such as BPA and EE2 can act either as acceptors or donors in H bond formation, which is supported by the inverse relationship between the Kow values of the compounds and their adsorption constants [52].

Although the adsorption efficiency of HC is more limited than that of other adsorbents, such as activated carbon or BC, its production and use are simple, eco-compatible and inexpensive, making HC attractive for wastewater treatment or immobilization of contaminants in soil.

Table 8 shows the main results of some studies on EE adsorption on HC, including the most appropriate kinetics and isotherm models and adsorption capacities. Most studies concern the adsorption of BPA on HC [49,52,56], which is undoubtedly due to the wide diffusion of BPA in both terrestrial and aquatic environments, although a significant number of studies concern StEEs. Some works investigated the sorption activity of unmodified HC [49,52,102], while others included a preliminary activation of HC [56].

In general, kinetics data of EEs on HC show a better correlation with the PSO model (Table 8), which suggests that chemisorption is the main adsorption mechanism involving prevalently π–π electron acceptor–donor bonds and electrostatic interactions. Differently, equilibrium isotherm data of EEs follow almost equally the Freundlich and Langmuir equations (Table 8), which indicates the importance of the type of HC considered. The Freundlich model yields the best correlation of adsorption data when the adsorbent has a heterogeneous surface and the solute forms a multilayer on the sorbent with multiple interactions among the solute molecules. The Langmuir isotherm is the most appropriate when the adsorbent has a homogeneous surface and the solute molecules do not interact with each other and form a monolayer on the adsorbent. Similar to what was observed in the studies on BC, the activation of HC does not appear to change the preferred isotherm model, compared to unmodified HC [58].

The sorption capacity of HC is quite variable as it greatly depends on the specific surface area, porosity and the number and type of functional groups present on HC. In a recent work, de Lima et al. [56] found a very high sorption capacity of BPA on a NaOH-activated HC. Likewise, a KOH-activated HC was very efficient in the removal of E2 and EE2 [57].

Like BC, HC, besides being applied for water treatment, can be used for soil remediation. In particular, HC added to soil can immobilize organic pollutants, especially those with high hydrophobicity, thus regulating their bioavailability for plants and microorganisms and limiting the leaching of the less hydrophobic ones. Recently, a pioneering study by Loffredo and Parlavecchia [37] tested HC, BC and spent coffee grounds to permanently remove some EDCs, including BPA and OP, from a loamy soil. Although in that study HC showed a lower removal capacity than BC, it clearly favored the subsequent mycodegradation of the contaminants more than BC [37].

The regeneration and reusability of adsorbents is a key criterion for practical applications in water treatment. This aspect was investigated by various researchers who tested different methodologies to separate the contaminants from the adsorbents. Common protocols adopted for the desorption of contaminants are based on the use of chemical agents, such as acids, bases, salts and organic solvents such as methanol and ethanol [56,67] or acetone [58]. Physical treatments, for example, a new HTC cycle [49], are also adopted for HC regeneration.

#### 6.2.3. Digestate

In parallel with the development of the AD technology for biowaste recycling and bioenergy production, there has been an increase in scientific efforts to study and valorize the solid and liquid byproducts of AD in compliance with environmental and economic sustainability. According to the Scopus database and searching in AT&A&K, of all DG-based studies of the past ~70 years (3644 documents), 93% were published in the last decade (queried on 26 January 2022). Italy ranks first in the world for scientific production concerning DG, followed by China, the United States and Germany. Most of the entire scientific production on DG concerns the AD process and its optimization, biogas production, the use of DG as soil fertilizer and new research trends in DG valorization [124]. A significant number of works in recent years have focused on the thermochemical conversion of DG to produce BC or HC for environmental applications [36] or the bio-oxidative conversion of DG to obtain compost and vermicompost for agricultural applications [125,126].

Although agriculture is currently the end-user of DG, some aspects of this material deserve to be investigated and valorized with a view to potential environmental benefits. In particular, when DG is incorporated into soil, besides improving soil fertility and contributing to carbon sequestration, it plays the important role of interacting with both natural and xenobiotic organic compounds present in soil, thus modulating their bioavailability and reducing the risk of leaching/transport of contaminants to natural waters. The presence of pollutants such as EEs in soil depends on agricultural practices such as irrigation with wastewater or addition of contaminated biomass. Studies published so far on the interaction of DG with organic contaminants are limited, and those on DG–EE interaction are almost lacking. However, this is an important topic given the increasing extent of contaminated agricultural land and the ability shown by DG to significantly retain pollutants of different hydrophobicity [12].

Compared to other materials originating from biowaste such as BC and HC, DG shows a lower but not negligible adsorption capacity. The adsorption efficiency of DG is strictly related to the raw biomass used and the AD parameters adopted (temperature, retention time, microbial consortia and so on), which, in turn, determine the composition, microstructure, content and type of functional groups of DG. Exploring and valorizing DG as an innovative biosorbent is economically and ecologically interesting if one considers that DG has long been considered a hazardous waste to be disposed of. The first work (on Scopus database) on the use of DG for decontamination purposes was carried out by Mukherjee et al. [61], who used soil/DG biomixtures to obtain biobeds and biofilters to immobilize pesticides with contrasting physicochemical properties. Yao et al. [62] used an unmodified DG from OFMW for the removal of dyes from textile wastewater and reported the satisfactory performance of this material, thus demonstrating that DG could be a promising renewable and cost-effective decontaminant. A very recent study on adsorption/desorption of EEs on/from DG demonstrated that this material has a remarkable efficiency in removing BPA and, especially, OP from water and that the occurrence of a positive hysteresis guarantees a long-lasting retention of the pollutants [12].

## 7. Conclusions

Global concerns about the entry of dangerous organic pollutants, such as phenolic and steroidal EEs, into surface and groundwater and the urgent need to reclaim and reuse wastewaters have prompted the search for new sustainable strategies. A promising approach would be the use of biosorbents originating from the thermochemical and biological conversion of biowaste. In particular, coproducts and byproducts of bioenergy production, such as BC, HC and DG, show a relevant ability to remove organic pollutants, including EEs, from water and contribute significantly to soil remediation. The efficiency of these materials is related to their physicochemical properties and the type of contaminant, in particular its hydrophobicity. This review discusses the main aspects of these biosorbents, EEs and the related adsorption process both as it regards the interaction mechanisms and the modeling of the sorption data which allows the quantitative evaluation of the process. The main analytical methods used for the characterization of biosorbents and the evaluation of EE–biosorbent adsorption are also described. The remediation approach presented in this review could in perspective integrate with the other currently available strategies as it represents a valid and sustainable alternative to more sophisticated and expensive methods. However, to realize large-scale applications of these biosorbents, further studies are needed to make them competitive in terms of performance. The most modern and advanced characterization techniques and further improvement of the activation treatments of the materials will be extremely useful to achieve this goal, with great benefits for the economy and the environment. Finally, considering that these materials are ‘waste from waste’ at negligible costs and their performance as biosorbents is constantly improving, their valorization for sustainable use in water treatment and soil remediation is certainly convenient. Furthermore, it appears useful to highlight that the production and use of these materials are in line with the modern approach to sustainable resource management, carbon sequestration, implementation of the circular economy and environmental remediation.

## Figures and Tables

**Table 1:** Operating conditions of biomass conversions that produce biosorbents. From the literature [30,33].

	Biochar			Hydrochar	Digestate
Type of biomass conversion	Thermochemical			Thermochemical	Biochemical
Process	Slow pyrolysis	Fast pyrolysis	Gasification	Hydrothermal carbonization	Anaerobic digestion
Type of feedstock	Agricultural residues Woody residues			Agricultural residues OFMSW	Agricultural residues Livestock wastes Sewage sludge OFMSW
Feedstock moisture	Dry			Wet	80–90%
Temperature (°C)	300–650	500–650	800–900	180–260	Psychrophilic (20–25) Mesophilic (35–37) Thermophilic (>55)
Residence time	1–12 h	<2 s	10–20 s	1–12 h	14–30 days (mesophilic) 14–16 (thermophilic)
Pressure	-	-	-	Autogenous (2–10 MPa)	-
Product yield (%)					
Solid	25–35	12	<10	50–80	-
Liquid	20–30	75	<5	5–20	-
Gases	25–35	13	>85	2–5	60–70 (fresh biomass)

**Table 2:** Analytical techniques adopted to characterize biosorbents.

Sample	Feedstock	Process T (°C)	Elemental Analysis	SEM, SEM- EDX	BET	XRF	FTIR	TG, DTG	NMR	Ref.
BC	Poultry litter and wheat straw	400	•		•		•		•	[52]
BC	Pinewood	500	•	•	•	•	•			[63]
BC	Corncob	700	•	•						[64]
BC	Swine solids and poultry litter	250, 450, 600	•	•	•				•	[53]
BC	Eucalyptus wood	600	•	•	•					[43]
BC	Digestate	Various (from 300 to 900)		•	•					[41]
BC	Dairy manure and sorghum	600	•	•	•		•			[37]
BC	Algae and sorghum	500	•	•	•		•			[38]
BC	Red spruce and grapevine wood	550	•	•		•	•	•		[32]
BC	Lotus seedpod	650	•	•	•		•			[44]
BC	Wheat straw	700		•	•		•			[40]
HC	Orange peels	200	•	•	•		•			[64]
HC	Swine solids and poultry litter	250	•	•	•				•	[53]
HC	Corncob	230	•	•						[64]
HC	Olive pomace	180–250	•				•	•		[65]
HC	Lignin	240, 300	•				•			[66]
HC	Rice husk	200		•	•		•			[58]
HC	Rice husk	180	•	•	•		•	•		[57]
HC	Urban pruning and OFMW	180–210	•	•		•	•	•		[32]
HC	Sewage sludge	160, 190, 250	•	•	•		•			[49]
HC	Argan nut shells	180, 200		•	•	•	•			[67]
HC	Pine fruit shells	190		•	•		•			[56]
DG	Swine manure	34	•				•	•	•	[68]
DG	Food waste	-	•	•			•			[8]
DG	Swine manure	-	•					•		[41]
DG	Mixed residues and olive pomace	20–45		•			•			[12]

BC: biochar; HC: hydrochar; DG: digestate; SEM and SEM-EDS: scanning electron microscopy and SEM coupled to energy-dispersive X-ray spectroscopy; BET: Brunauer–Emmett–Teller analysis; XRF: X-ray fluorescence spectroscopy; FTIR: Fourier transform infrared spectroscopy; TG and DTG: thermogravimetric and derivative thermogravimetric analysis; NMR: nuclear magnetic resonance spectroscopy.

**Table 3:** Some results of biochar (BC), hydrochar (HC) and digestate (DG) characterization.

	Process	Feedstock	Process T (°C)	Residence Time	Elemental Composition ^a^ (%)				pH ^b^	EC ^b^ (dS m^−1^)	Ash (%)	Surface Area ^c^ (m^2^ g^−1^)	Avg. Pore Size (nm)	Pore Volume (cm^3^ g^−1^)	Ref.
					C	H	N	O							
BC	Py	Digestate from swine manure	800	1.3 h	--	--	--	--	--	--	--	101.9	3.04	0.08	[41]
BC	Py	Algae	500	2 h	24.6	1.3	3.2	11.4	10.2	10.70	59.7	0.5	1.88	0.16	[38]
BC	Py	Sorghum	500	2 h	46.7	3.0	0.0	13.0	7.4	5.95	29.4	4.1	13.22	13.27	
BC	Py	Sorghum	600	2 h	47.4	2.3	0.0	9.8	9.6	5.92	45.1	4.1	13.29	12.99	
BC	Py	Red spruce wood	550	3 h	84.0	1.5	0.2	n.d.	9.1	0.39	4.7	--	--	--	[32]
BC	Py	Grapevine pruning	550	3 h	75.5	1.3	0.5	n.d.	9.9	2.23	9.9	--	--	--	
BC	Py	Lotus seedpod	650	2 h	69.9	2.1	1.1	15.6	--	--	--	25.2	2.55	0.03	[44]
*KOH*- BC	Py	Lotus seedpod	650	2 h	78.8	2.4	1.3	15.1	--	--	--	306.2	1.90	0.13	
BC	Py	Wheat straw	700	2 h	--	--	--	--	--	--	--	57.2	3.70	0.05	[40]
*NaOH*-BC	Py	Wheat straw	700	2 h	--	--	--	--	--	--	--	254.9	1.92	0.12	
*HCl*- BC	Py	Wheat straw	700	2 h	--	--	--	--	--	--	--	197.2	2.86	0.14	
HC	HTC	Pig manure	200	2 h	33.8	4.2	2.5	15.0	8.3	19.86	44.0	--	--	2.10	[39]
HC	HTC	Pig manure	240	2 h	25.8	3.0	1.9	10.4	7.8	10.93	58.6	--	--	2.80	
HC	HTC	Urban pruning	210	8 h	61.5	6.2	1.7	--	6.6	1.03	12.5	--	--	--	[32]
HC	HTC	OFMSW	210	8 h	62.6	6.0	1.7	--	7.7	1.09	15.7	--	--	--	
HC	HTC	Sewage sludge	160	4 h	30.8	4.9	3.2	14.0	5.1	6.10	46.5	9.5	--	--	[49]
HC	HTC	Sewage sludge	190	4 h	30.0	4.3	2.4	11.4	5.7	8.44	51.3	11.9	--	--	
HC	HTC	Sewage sludge	250	4 h	31.0	4.1	2.4	8.00	6.6	16.53	53.8	2.9	--	--	
DG	AD	Food waste	--	--	42.1	5.2	5.8	21.3	--	--	25.6	--	--	0.32	[8]
DG	AD	Mixed residues	--	30 d	40.0 ^d^	--	6.5	--	8.7	--	--	3.10	--	--	[61]
DG	AD	Swine manure	--	--	37.2	5.5	4.6	31.9	--	--	23.0	--	--	--	[41]
DG	AD	Mixed residues	30	40 d	50.5 ^d^	-	-	-	8.7	1.36	12.8	--	--	--	[12]

Py: pyrolysis; HTC: hydrothermal carbonization; AD: anaerobic digestion; ^a^ on dry and ash-free basis; ^b^ 1:10 (*w*/*v*) in double-distilled water; ^c^ calculated by the BET method; ^d^ total organic C; --: not reported.

**Table 4:** Chemical structure and physicochemical properties of phenolic and steroidal estrogens (EEs).

EE	Chemical Structure	Molecular Weight (g mol^−1^)	Water Solubility (mg L^−1^ at 25 °C)	LogKow	pKa	Vapor Pressure (mm Hg at 25 °C)
BPA	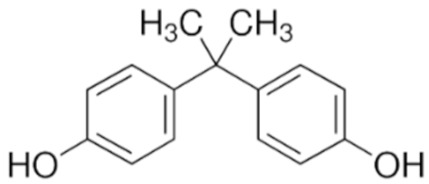	228.29	300	3.32	9.6	4.00 × 10^−8^
4-tert-OP	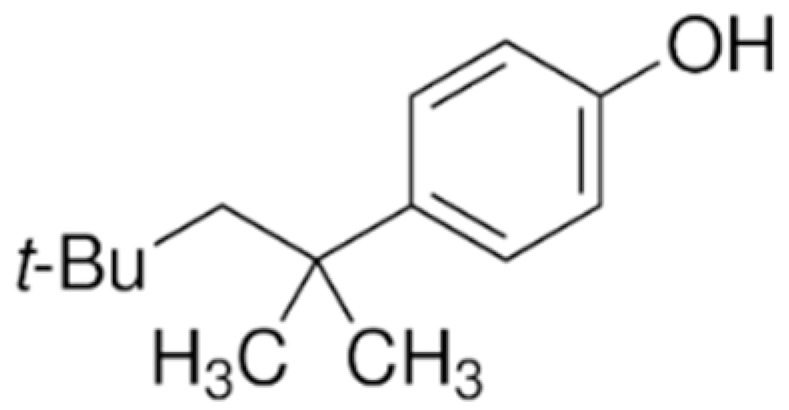	206.32	5.1	5.25	10.33	4.80 × 10^−4^
NP	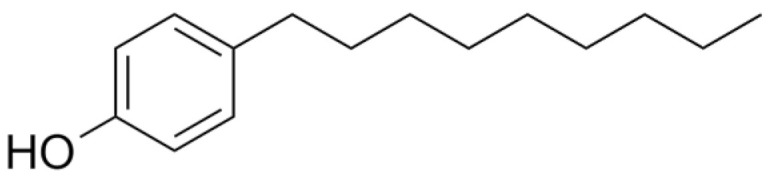	220.35	7.0	5.76	10.31	8.18 × 10^−4^
E1	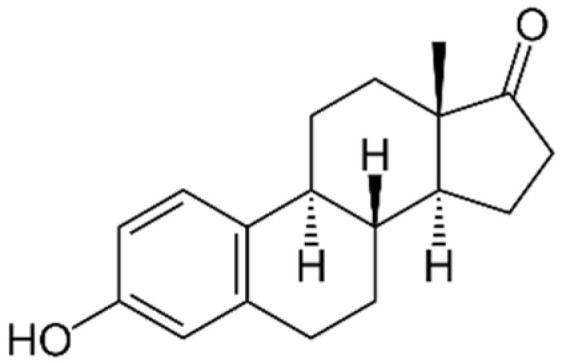	270.37	30	3.13	10.40	2.49 × 10^−10^
E2	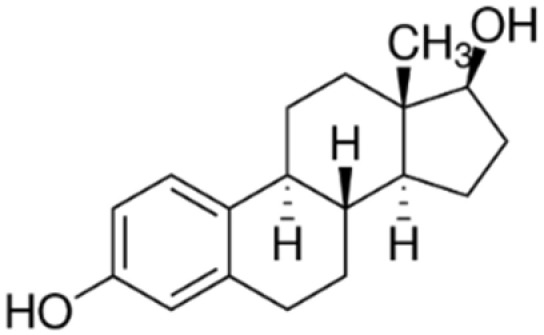	272.38	3.9	4.01	10.71	6.38 × 10^−9^
E3	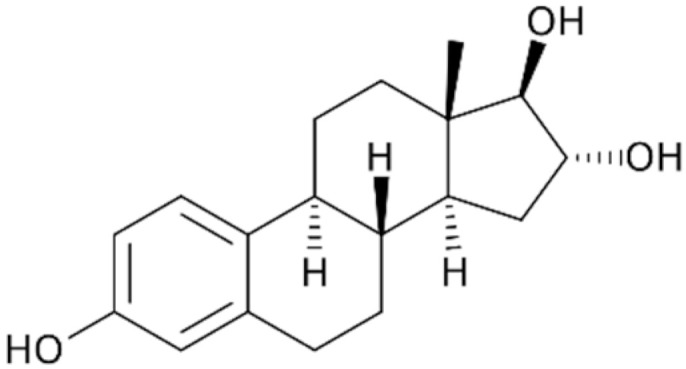	288.39	27.3	2.45	10.33	9.93 × 10^−12^
EE2	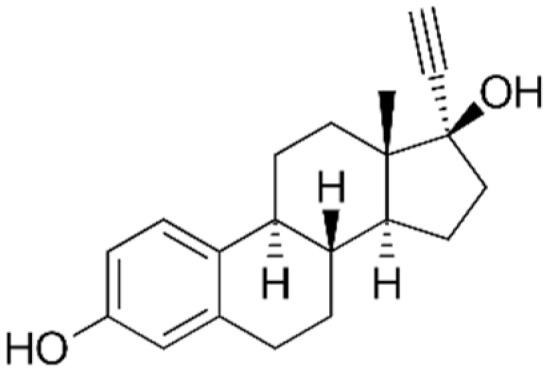	296.41	11.3	3.67	10.33	1.95 × 10^−9^

Data from PubChem [75].

**Table 5:** Methods for the removal of environmental estrogens (EEs) from water and soil.

Method	Advantages	Limitations	EE	Ref.
Water				
Immobilization				
Coagulation/sedimentation	High efficacy for the elimination of EEs having a logKow > 4		BPA, NP, E1, E2, E3	[26]
Destruction				
Chemical and Photochemical Treatments				
Advanced oxidation processes (AOPs) Photocatalysis using catalysts and nanocatalysts (TiO_2_, ZnO and others) Electrochemical oxidation	High efficacy Low environmental impact Reduce estrogenic activity	High costs Ex situ application Incomplete elimination Complexity	BPA, NP, E1, E2, E3	[26] [27] [88]
Ozonation	Used in tertiary treatments, increases the efficacy of other methods	May generate byproducts more toxic and persistent than the parent compound	BPA, NP, E1, E2, E3	[26]
Chlorination	Used in tertiary treatments, increases the efficacy of other methods	May generate byproducts more toxic and persistent than the parent compound	BPA, NP, E1, E2, E3	[26]
UV photolysis	High efficacy for StEE removal Enhancement of the efficacy of other methods	Ineffective for the removal of other pollutants coexisting with EEs	BPA, NP, E1, E2, E3	[26]
Biological Treatments				
Activated sludge treatment	High efficacy		BPA, NP, E1, E2, E3	[26]
Membrane bioreactor	High removal efficacy Wide diversity of degrading microbial community	High retention time	BPA, NP, E1, E2, E3	[26]
Separation				
Ultrafiltration	High efficacy when used in combination with other methods and for hydrophobic compounds		BPA, NP, E1, E2, E3	[26]
Adsorption				
Use of activated carbon	High efficacy	High cost		
Use of recalcitrant carbon-rich biosorbents (biochar, hydrochar, digestate)	Innovative approach Low cost and simplicity Very effective for hydrophobic compounds	Lower sorptive performance than other expensive adsorbents	BPA, OP	*
**Soil**				
Containment				
Engineering techniques for the isolation of polluted sites and sources of contamination (containment barriers)	Reduced leaching/transport into natural waters	High costs High environmental impact High contaminant persistence		[89]
Immobilization				
Incorporation of recalcitrant carbon-rich materials, especially carbonaceous adsorbents, as soil amendments (biochar, hydrochar, digestate, compost)	Reduced leaching/transport Low cost and simplicity Very effective for highly hydrophobic compounds Reduced bioavailability Increased soil fertility	Possible desorption for less hydrophobic compounds Possible degradation of not sufficiently recalcitrant adsorbents	BPA, OP, E1 EE2	[90] [51] [91] [92] [93]
Destruction				
Chemical remediation				
Advanced oxidation processes (AOPs, Fenton processes, TiO_2_ photo-catalysis, ozonation, electrochemical oxidation	Mineralization of contaminants	High costs Ex situ application Incomplete elimination Complexity		[89]
Biological remediation				
Phytoremediation (plants and algae) Assisted phytoremediation (combination of plants and soil amendments)	Absorption by the root system and complete elimination by trans- formation in plant tissues Low cost and simplicity Environmentally friendly Increased soil fertility Less toxicity for plants using assisted phytoremediation	Long-lasting process Use of plants resistant to contaminants Not suitable for heavily contaminated sites		[89] [94]
Bioremediation (bacteria and fungi)	Absorption by microbial cells and biodegradation Low cost and simplicity Environmentally friendly Reduced bioavailability	Selection and inoculation of appropriate consortia of microorganisms or single strains		[89]
Separation				
Adsorption on carbon-rich biosorbents without incorporation in soil (biochar, hydrochar, digestate, compost)	Significant removal Low cost and simplicity Environmentally friendly	Incomplete removal Needs further study	BPA, OP	[37] [89]
Ex situ soil washing (extractants)	Complete elimination of contaminants	High costs High environmental impact Not suitable for all soil textures		[89]

* See references reported in tables of Section 6.2.

**Table 8:** Removal of different environmental estrogens (EEs) from water using hydrochar and digestate.

EE	Feedstock	Process T (°C)	Residence Time (h)	Activation	Kinetics Model	Isotherm Model	Adsorption Capacity (mg g^−1^) *	Ref.
Hydrochar								
BPA	Poultry litter	250	20	-	n. e.	F	77.62	[52]
BPA	Swine solids	250	20	-	n. e.	F	33.11	[52]
BPA	Magonia pubescens wood	180	6	-	PFO	L	21.26	[102]
BPA	Sewage sludge	160, 190, 250	4	-	n. e.	L	From 10.86 to 18.37	[49]
BPA	Argan nut shells	200	6	-	PSO	L	1162.79	[67]
BPA	Pine fruit shells	190	24, 48, 72	NaOH	PSO	L	From 332.52 to 378.77	[56]
E1	Swine solids	250	8	-	n. e.	F	100.48	[53]
E1	Poultry litter	250	8	-	n. e.	F	51.88	[53]
E2	Rice husk	200	6	-	n. e.	F	15.87	[58]
E2	Rice husk	200	6	KMnO_4_ + FeCl_2_	PSO	F	22.31	[58]
E2	Montmorillonite and rice husk	180	16	KOH	PSO	L	138.00	[57]
EE2	Swine solids	250	20	-	n. e.	F	18.62	[52]
EE2	Poultry litter	250	20	-	n. e.	F	181.97	[52]
EE2	Montmorillonite and rice husk	180	16	KOH	PSO	L	69.00	[57]
Digestate								
BPA	Mixed residues and olive pomace	20–45	28 days	-	PFO/PSO	F	0.13	[12]
OP	Mixed residues and olive pomace	20–45	28 days	-	PFO	F	1.07	[12]

* Data refer to K_F_ or Q_max_ based on preferred Freundlich or Langmuir isotherm, respectively, at temperature of 25 ± 2 °C; K_F_: Freundlich constant; L: Langmuir isotherm; F: Freundlich isotherm; PFO: pseudo-first order; PSO: pseudo-second order; n. e.: not evaluated.
